# Restoration of T Cell function in multi-drug resistant bacterial sepsis after interleukin-7, anti-PD-L1, and OX-40 administration

**DOI:** 10.1371/journal.pone.0199497

**Published:** 2018-06-26

**Authors:** Lukose K. Thampy, Kenneth E. Remy, Andrew H. Walton, Zachery Hong, Kelilah Liu, Rebecca Liu, Victoria Yi, Carey-Ann D. Burnham, Richard S. Hotchkiss

**Affiliations:** 1 Department of Anesthesiology, Washington University School of Medicine, St. Louis, Missouri, United States of America; 2 Department of Pediatrics-Division of Critical Care Medicine, Washington University School of Medicine, St. Louis, Missouri, United States of America; 3 Department of Immunology and Pathology, Washington University School of Medicine, St. Louis, Missouri, United States of America; 4 Department of Medicine, Washington University School of Medicine, St. Louis, Missouri, United States of America; 5 Department of Surgery, Washington University School of Medicine, St. Louis, Missouri, United States of America; Ohio State University Wexner Medical Center, UNITED STATES

## Abstract

**Background:**

Multidrug resistant (MDR) bacterial pathogens are a serious problem of increasing importance facing the medical community. MDR bacteria typically infect the most immunologically vulnerable: patients in intensive care units, patients with extensive comorbidities, oncology patients, hemodialysis patients, and other immune suppressed individuals are likely to fall victim to these pathogens. One promising novel approach to treatment of MDR bacteria is immuno-adjuvant therapy to boost patient immunity. Success with this strategy would have the major benefit of providing protection against a number of MDR pathogens.

**Objectives:**

This study had two main objectives. First, immunophenotyping of peripheral blood mononuclear cells from patients with sepsis associated with MDR bacteria was performed to examine for findings indicative of immunosuppression. Second, the ability of three immuno-adjuvants with distinct mechanisms of action to reverse CD4 and CD8 T cell dysfunction, a pathophysiological hallmark of sepsis, was evaluated.

**Results:**

Septic patients with MDR bacteria had increased expression of the inhibitory receptor PD-1 and its ligand PD-L1 and decreased monocyte HLA-DR expression compared to non-septic patients. All three immuno-adjuvants, IL-7, anti-PD-L1, and OX-40L, increased T cell production of IFN-γ in a subset of septic patients with MDR bacteria: IL-7 was most efficacious. There was a strong trend toward increased mortality in patients whose T cells failed to increase IFN-γ production in response to the three treatments.

**Conclusion:**

Immuno-adjuvant therapy reversed T cell dysfunction, a key pathophysiological mechanism in septic patients with MDR bacteria.

## Introduction

Over the past decade, there has been a dramatic increase in the incidence of severe infections due to multidrug resistant (MDR) bacteria, frequently termed “superbugs” [1–8]. In the United States and Europe, MDR bacteria are associated with more than 4 million infections and greater than 50,000 deaths, while causing an excess of 20 billion dollars in hospital costs [9,10]. Further, the number and diversity of resistant pathogens has been increasing due to continued widespread use and misuse of antibiotics. As the incidence of these organisms has increased, the number of effective antibiotics has consequently decreased, creating a very real threat that future bacterial infections will resist all modern methods of treatment. As such, the World Health Organization (WHO) has recently identified MDR pathogens as one of the most serious health care problems currently facing the public [11].

Multidrug resistant bacteria, with few exceptions, are largely indolent organisms which establish infection in the immunocompromised, critically-ill patient: patients with protracted hospitalization in intensive care units (ICUs), long-term residence in nursing homes, oncologic diseases, neonatal diseases of prematurity, burns, organ transplant, or chronic hemodialysis frequently succumb to these bacteria. These vulnerable populations are predisposed to sepsis, which further impairs immunity and drives mortality due to these normally harmless organisms. To this end, one emerging paradigm to treating MDR bacterial infections focuses on restoring and augmenting host immunity. Several independent studies in animals have demonstrated that a number of diverse immuno-adjuvants improve survival in clinically relevant models of sepsis, including sepsis associated with MDR pathogens [12, 13]. The authors found that boosting the immune system in a T cell dependent manner improved bacterial clearance and reversed sepsis-induced immunosuppression. Recently, clinical trials of immuno-adjuvants including interleukin-7 (IL-7), granulocyte macrophage colony stimulating factor (GM-CSF), thymosin α_1_, and the checkpoint inhibitors, anti-programmed cell death protein 1 (α-PD-1) and anti-programmed cell death ligand 1 (α-PD-L1), have been initiated in sepsis [14]. Thus, in addition to improved antibiotic stewardship, this new paradigm of boosting the immune system in the context of recalcitrant infection may ameliorate the emerging public health threat that MDR pathogens pose. If this strategy is proven as efficacious, it has the potential to change the approach to infectious disease and in critical illness in much the same way that immunotherapy with checkpoint inhibitors has revolutionized the field of oncology.

Thus, we conducted a study to determine if immuno-adjuvant therapy could reverse impaired T cell effector function present in patients with sepsis associated with MDR pathogens. There is extensive evidence that sepsis causes profound loss of CD4 and CD8 T cells; surviving T cells are poorly functional and exhibit an “exhausted” phenotype that mimics pathophysiological features seen in malignancy [15]. We tested three immuno-adjuvants: interleukin-7 (IL-7), OX-40 ligand (OX-40L), and anti-programmed cell death ligand 1 (anti-PD-L1). These three immuno-adjuvants were selected, as their primary cellular sites of action are CD4 and CD8 T cells, and are either currently clinically approved or actively in use in ongoing clinical trials for immunotherapy of sepsis or oncology [12–19 and NCT02797431, NCT02960854, NCT02274155]. Not only do all three agents have reported efficacy in animal infectious models, but IL-7 and anti-PD-1 reverse T cell dysfunction in ex vivo blood samples from patients with sepsis [16–19]. Immunophenotyping of CD4 and CD8 T cells and monocytes was performed to quantitate expression of immunologic markers associated with immunosuppression in patients with sepsis.

## Materials and methods

### Study design

We conducted a prospective study among patients with MDR sepsis compared to critically-ill non-septic patients (CINS or control) cared for in a mixed medical and surgical ICU between 2016 and 2017 at Barnes-Jewish Hospital (St. Louis, MO). Analyses were performed on residual EDTA-blood specimens following routine clinical hematology laboratory testing.

#### Inclusion criteria

Hospitalized patients, 18 years old or greater, who had sepsis as defined by having 2 or more criteria for systemic inflammatory response syndrome (SIRS) and a clinically or microbiologically suspected infection were included in the study [20]. Patients also had to have either a positive blood culture (n = 22 patients) or a bronchoalveolar (BAL) lavage (n = 2 patients) with any of the following multi-drug resistant pathogens including: *Enterococcus spp*., methicillin-resistant *Staphylococcus aureus* (MRSA), *Pseudomonas aeruginosa*, *Stenotrophomonas maltophilia*, *Acinetobacter calcoaceticus*-*baumanii* complex, or *Klebsiella spp* These pathogens were selected because they are commonly associated with sepsis in ICU patients and have been identified by the Centers for Disease Control (CDC) as emerging threats due to increasing antimicrobial resistance [21].

#### Exclusion criteria

To minimize confounding effects of immunosuppressive medications or underlying immunologic disease on the findings from the present investigation, patients with the following criteria were excluded:

Patients with HIV, organ or bone marrow transplantation, use of current high-dose corticosteroid regimens that were greater than or equivalent to 300 mgs/day of hydrocortisone or other immunosuppressive medications, current use of immune-modifying biological agents including inhibitors of TNF-α or other cytokines, viral hepatitis, or systemic autoimmune diseases.

#### Flow cytometry

Expression levels of phenotypic markers consistent with immunosuppression were determined via flow cytometry. These samples were analyzed for both percent of cells positive for marker expression and for geometric mean fluorescence intensity (GMFI), a quantitative measure of the expression levels of receptors or ligands expressed on an individual cell (per cell basis). Examination of phenotypic markers associated with T cell exhaustion included programmed cell death 1 (PD-1, CD279) and IL-7 receptor α (CD127). T cell activation, measured by CD4 and CD8 expression of OX-40 (CD134), was also investigated. Last, we quantitated monocyte expression of HLA-DR and PD-L1.

Antibodies employed were as follows: CD3-FITC, CD4-Per_CP/Cy5.5, CD8-APC_Cy7, PD1-PE, OX-40-APC, IL-7 receptor α (CD127-APC), PD-L1-PE, neutrophil marker CD15-FITC (BioLegend, San Diego, CA), and Quantibrite Anti-HLA-DR/Anti-Monocyte_PerCP/Cy5.5 (BD, Franklin Lakes, NJ). See S1 Table for clones/catalog numbers.

Cell preparation for flow cytometry was performed as previously described [22]. Briefly, whole blood was stained, followed by a hypotonic RBC lysis buffer (BioLegend, San Diego, CA), and subsequent fixation in 1% paraformaldehyde. HLA-DR surface marker was quantitated as previously described [23]. All samples were acquired on a FACScan^™^ (BD Biosciences, San Jose, CA, USA) System upgraded to five colors (Cytek Biosciences, Fremont, CA, USA), and analyzed by FlowJo (version 10.0.8r1). Lymphocytes were identified by forward scatter (FSC) and side scatter (SSC) properties and by CD3+, CD4+, or CD8+ immunostaining.

#### ELISpot quantitation of T cell IFN-γ production

IFN-γ is secreted by CD4 and CD8 T cells and is a potent activator of monocytes and macrophages, key components of the innate immune system that play an essential role in pathogen killing. Importantly, loss of T cell IFN-γ production is a hallmark of T cell exhaustion and has been shown to correlate with mortality in animal models of sepsis [24]. This study tested the ability of three immuno-adjuvants with differing mechanisms of action to increase T cell IFN-γ production. Quantitation of the number of IFN-γ producing T cells was assessed in peripheral blood mononuclear cells (PBMCs) by ELISpot analysis as per the manufacturer’s instruction (R&D Systems). Patient PBMCs were harvested from whole blood via Ficoll-Paque^™^, plated at a standardized density, 5 x 10^5^ cells per well using the Vi-Cell^™^ counter from Beckman Coulter (Brea, CA, USA), and incubated overnight with RPMI 1640 media (Sigma-Aldrich, St. Louis, MO)—supplemented with human AB serum, non-essential amino acids, penicillin/streptomycin, and glutamine—containing anti-CD3/anti-CD28 (BioLegend, San Diego, CA) as stimulants. The means of two identically treated plates run in duplicate were calculated.

Immuno-adjuvants were obtained from R&D Systems with the following catalog numbers: 207-IL-200, AF156, and 1054-OX. Each ELISpot well contained a volume of 200 μl with final concentrations of IL-7, anti-PD-L1, and OX-40 ligand of 50 ng/ml, 10 μg/ml and 100 ng/ml, respectively. The isotype control antibody was from R&D Systems (catalog #AB108-C). ELISpot plates were made by Merck Millipore and obtained through Fisher Scientific (Hampton, NH; catalog number M8IPS4510). IFN-γ was detected using a colorimetric reagent kit (Strep-AP and BCIP-NBT, R&D Systems, Minneapolis, MN, USA; catalog number SEL002). Following development, images were captured and analyzed on Cellular Technologies Ltd (Cleveland, OH) ImmunoSpot 7.0 plate reader and software.

#### Data and sample collection

Remnant blood specimens from patients with bloodstream infections were typically obtained 24–48 hours of cultures returning positive for MDR bacteria. Patient survival was followed for 90 days after study entry.

#### Statistical methods

All statistical analyses were performed using GraphPad Prism 6.0 (San Diego, CA) and IBM SPSS statistics v25. Normality of each data set was tested by histogram followed by the Pearson chi square test. If data met normality criteria, a Student’s t-test with Welch’s correction was employed to assess statistical significance. Data not meeting these criteria were analyzed via Mann-Whitney test. Comparisons of differences in continuous variables within a group (isotype control vs treatments) were done using paired Student t-tests, one way ANOVA and multivariate analysis. P-values of <0.05 were considered significant.

#### Human study ethics statement

The Washington University Institutional Review Board/Human Research Protection Office granted this study a waiver of informed consent for obtaining excess clinical “waste” laboratory blood (which was due to be discarded), and for review of their relevant hospital records. These procedures were considered to represent minimum risk to the patients per 45CFR 46.102(i) and/or 21 CFR56.102(i) as applicable. The IRB identification number for this protocol is 201102561.

## Results

### Clinical parameters and disease course

24 patients with sepsis and specimens positive for MDR bacteria and 26 critically-ill non-septic control patients (CINS) were enrolled (Table 1). The mean age of the patients was 61.5 years (range 30–91) and 59.5 (range 20–84) for the septic and CINS cohorts, respectively (p = 0.68). The MDR sepsis cohort had more comorbidities vs the CINS cohort, including increased incidence of diabetes, renal disease, respiratory disease, and cancer. 90-day mortalities were 37.5% and 0% in the MDR cohort and the CINS cohort, respectively. The identification of the bacterial pathogens included the following: *Enterococcus spp*. (n = 10), MRSA (n = 6), *Acinetobacter calcoaceticus-baumanii* (2), *Pseudomonas aeruginosa* (n = 5), *Stenotrophomonas maltophilia* (n = 2), and *Klebsiella spp*. (n = 8) (Table 2). 10 MDR septic patients tested positive for more than one bacterial species during their stay in the ICU.

**Table 1:** Clinical characteristics of patients.

	Sepsis + MDR Bacteria (n = 24)	Critically-Ill, Non-Septic (CINS) (n = 26)
Age (mean ± SEM)	61.5±3.5	59.5±3.1
Male/Female	14/10	16/10
90-day mortality	37.50%	0%
**Comorbidities**:		
Cancer	5(20.8%)	0(0%)
Diabetes	9(37.5%)	4(15.4%)
Heart disease	14(58.3%)	17(65.4%)
Morbid obesity	2(8.3%)	3(11.5%)
Renal disease	12(50%)	4(15.4%)
Neurologic disease	10(41.7%)	12(46.2%)
Respiratory disease	15(62.5)	4(15.4%)
**Primary Diagnosis**	• Sepsis/Septic Shock (n = 5, 20.8%)	• Trauma/Hemorrhage (n = 15, 57.7%)
	• Trauma/Hemorrhage (n = 5, 20.8%)	• Atrial Fibrillation (n = 3, 11.5%)
	• Respiratory disease (n = 4, 16.7%)	• Esophageal disease (n = 2, 7.7%)
	• Cancer (n = 4, 16.7%)	• Spinal complications (n = 2, 7.7%)
	• Biliary injury (n = 1, 4.2%)	• Respiratory failure (n = 2, 7.7%)
	• Coronary Artery Disease (n = 1, 4.2%)	• Aneurysm (n = 1, 3.8%)
	• End Stage Renal Disease (n = 1, 4.2%)	• Syncope (n = 1, 3.8%)
	• Delirium (n = 1, 4.2%)	
	• Hypopituitarism (n = 1, 4.2%)	
	• Deep Vein Thrombosis (n = 1, 4.2%)	

**Table 2:** MDR species in septic patients.

**Microbiology**	*Enterococcus spp*. (10)
	*Klebsiella spp*. (8)
	*Methicillin-Resistant Staphylococcus Aureus* (6)
	*Pseudomonas Aeruginosa* (5)
	*Stenotrophomonas Maltophilia* (2)
	*Acinetobacter calcoaceticus-baumanii* (2)
**Foci of positive cultures**	Bloodstream (22)
	BAL (2)

**Fig 1:**
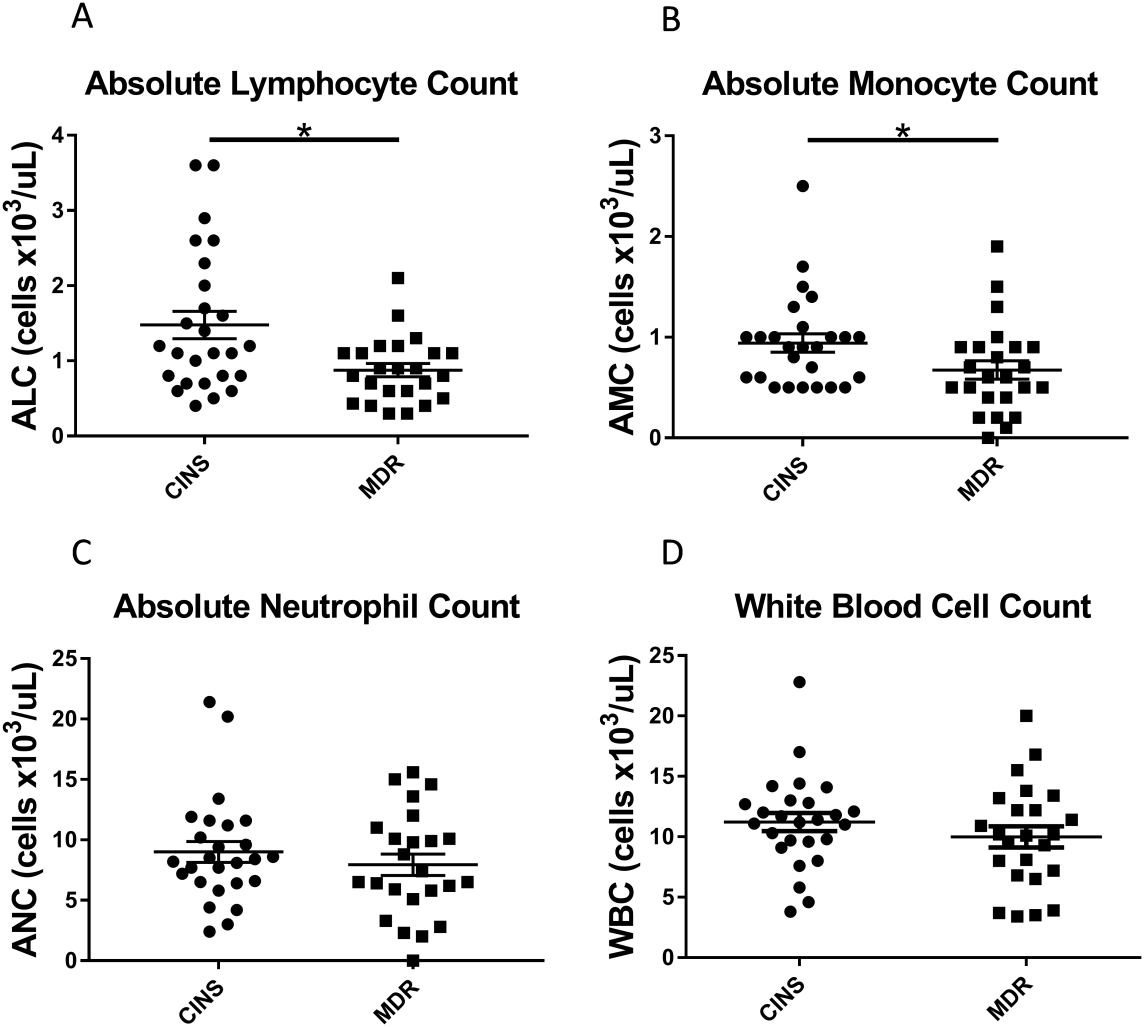
Quantified mean white blood cells. Cell counts (cells x 10^3^/μL) are presented as Absolute Lymphocyte Count (a), Absolute Monocyte Count (b), Absolute Neutrophil Count (c) and White Blood Cell count (d) in Multidrug Resistant (MDR) Septic patients compared to Critically-Ill Non-Septic (CINS) patients. Bars represent mean +/- S.E.M. *p<0.05.

There was no difference in either the total white blood cell count or absolute neutrophil count in the MDR septic vs CINS cohorts (*p* = 0.30 and *p* = 0.40, respectively; Fig 1). In contrast, the absolute lymphocyte count, which correlates with survival in sepsis [25], was significantly lower in MDR septic patients vs CINS patients (0.88±0.09 vs 1.48±0.18 x 10^3^ cells/μL; *p* = 0.005, Fig 1). Similarly, the absolute monocyte count was significantly lower in septic patients compared to CINS patients (0.68±0.09 vs 0.94±0.09 x 10^3^ cells/μL; *p* = 0.04, Fig 1).

### Flow cytometry: Evidence of T cell exhaustion and impaired monocyte antigen presentation

**Fig 2:**
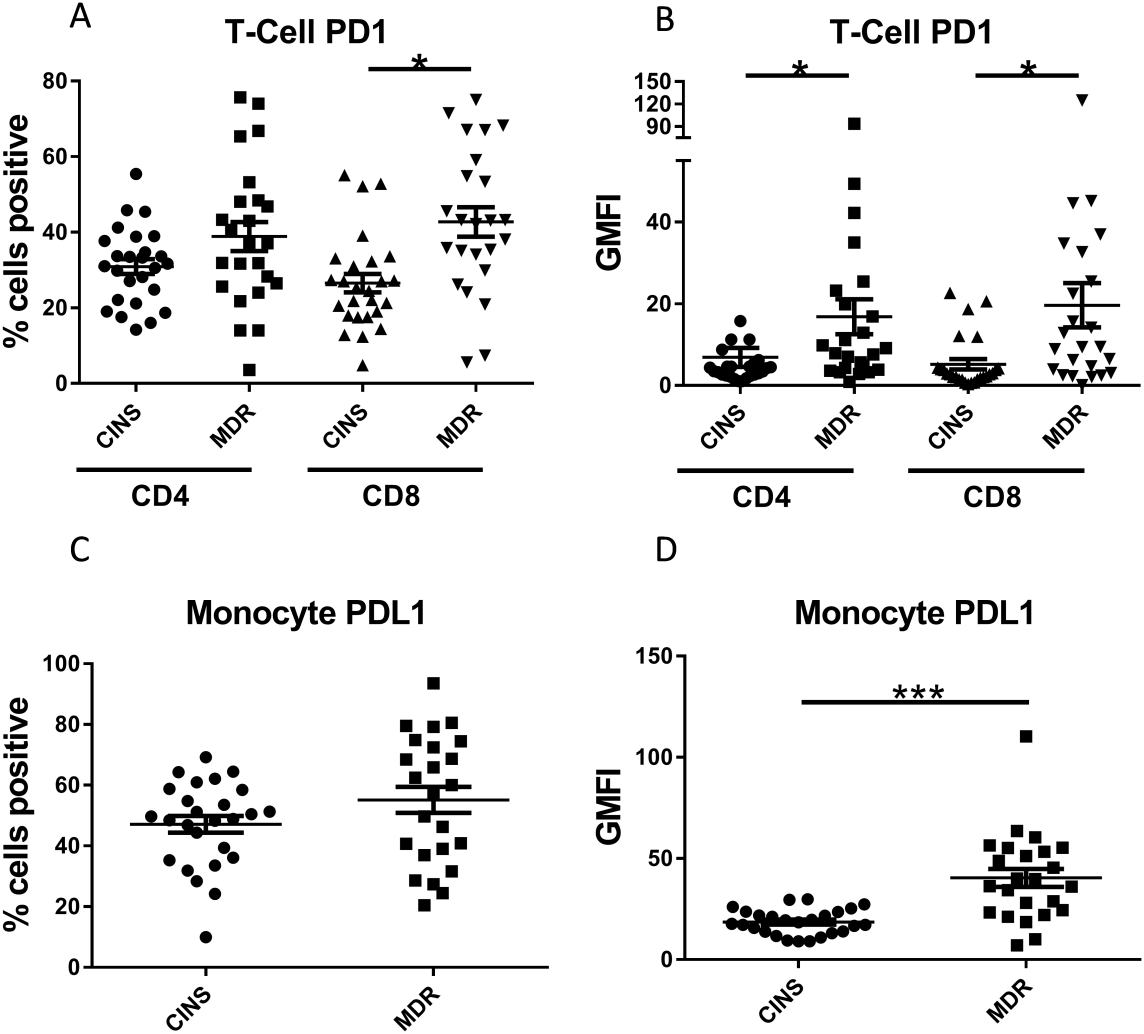
Flow cytometric expression of PD-1 and PD-L1. PD-1 was increased in patients with sepsis attributed to Multidrug Resistant (MDR) bacteria compared to Critically-Ill Non-Septic (CINS) patients on both CD4 and CD8 T cells, both in the percentage of cells bearing the receptor (a; % cells positive) and the intensity of staining (b; GMFI). GMFI = geo mean fluorescence intensity. Surface PD-L1 on monocytes showed increased staining intensity (d), however, there was insufficient evidence of a significant increase in the percentage of PD-L1 positive monocytes (c). Bars represent mean +/- S.E.M. *p<0.05, ** p<0.01, ***p<0.001.

First, we examined inhibitors of T cell cytokine production and proliferation via cellular receptor PD-1 upon binding its corresponding ligand (PD-L1). The percentage of CD4 and CD8 T cells expressing PD-1 in MDR septic patients was increased as compared to CINS control patients, 38.87±3.84% vs 29.95±1.79% and 42.75±3.90% vs 27.09±2.48%, respectively, (*p* = 0.075 and p = 0.001, respectively, Fig 2A, Table 3). Similarly, the GMFI of PD-1 on individual CD4 and CD8 T cells on a per cell basis (geometric mean fluorescence intensity; GMFI), was also increased in MDR septic patients vs CINS patients (16.83±4.28 vs 4.67±0.71 and 19.62±5.42 vs. 4.92±1.27, *p* = 0.049 and *p* = 0.015, Fig 2B, Table 3). The percentage of monocytes expressing PD-L1 trended higher in MDR septic individuals (55.14± 4.3% vs 47.11± 2.75%, *p* = 0.124), this relationship did not reach statistical significance. However, the GMFI of PD-L1 on monocytes in MDR patients was significantly higher compared to monocytes from non-septic counterparts (40.4±4.47 vs 18.57±1.22, *p*<0.0001).

**Table 3:** Flow cytometric data.

		*% Positive*		GMFI	
Marker	Cell Type	Control	Septic	Control	Septic
OX-40	CD4	17.18(+/-1.3)	14.69(+/-2.11)	8.51(+/-0.74)	10.35(+/-2.11)
	CD8	1.72(+/-0.28)	2.95(+/-0.85)		
PD-L1	CD4	29.95(+/-1.79)	38.87(+/-3.84)*	4.67(+/-0.71)	16.83(+/-4.28)*
	CD8	27.09(+/-2.48)	42.75(+/-3.9)*	4.92(+/-1.27)	19.62(+/-5.42)*
IL-7Rα	CD4	78.8(+/-2.07)	69.97(+/-4.15)	54.88(+/-2.79)	45.56(+/-4.68)
	CD8	45.13(+/-5.18)	48(+/-5.09)	38.05(+/-5.47)	33.98(+/-4.01)
PD-L1	Monocytes	47.11(+/-2.75)	55.14(+/-4.3)	18.57(+/-1.22)	40.4(+/-4.47)***
HLA-DR	Monocytes			26811.35(+/-2809.3)	16217.77(+/-2507.43)

*p<0.05,

**p<0.01,

***p<0.001

**Fig 3:**
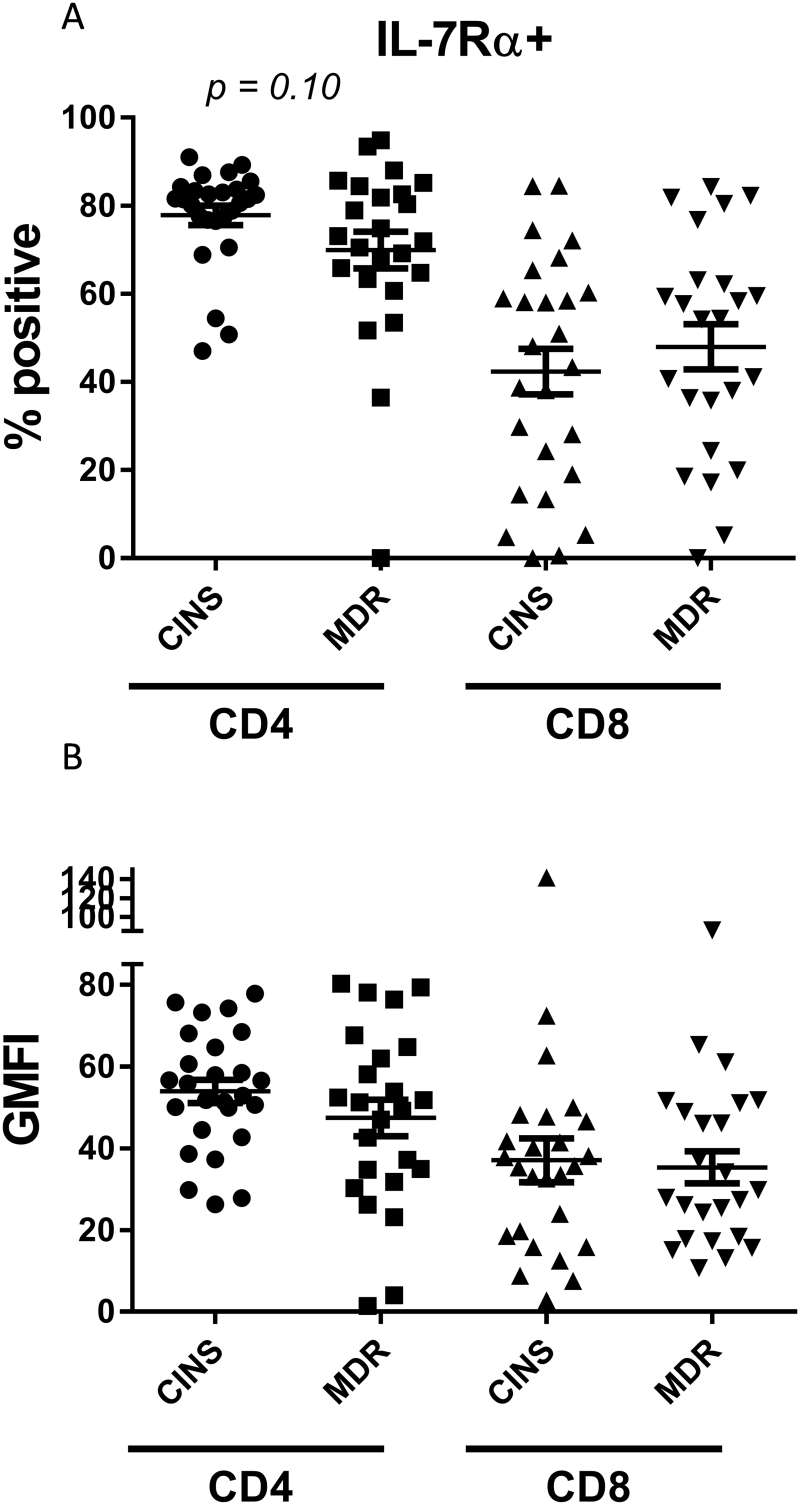
IL-7Rα surface levels on T cells. There was a modest non-significant decrease in CD127 (IL-7Rα) positive CD4 T cells (*p* = 0.1) (a). There was no difference in IL-7Rα positive CD8 T cells (a). There was also no change in receptor intensity on either CD4 or CD8 T cells (b). Bars represent mean +/- S.E.M.

Next, we examined markers of T cell exhaustion including IL-7 receptor α (CD127, IL-7Rα) expression [26]. The percentage of CD4 T cells expressing IL-7Rα in MDR patients trended lower than those from CINS patients (69.97±4.15 vs 78.8±2.07, *p* = 0.10; Fig 3a), although this relationship did not reach statistical significance. Similarly, there was a non-statistically significant trend toward decreased GMFI of IL-7R on CD4 T cells of MDR septic patients vs CINS patients (45.56±4.68 vs 54.88±2.79; Fig 3b). There was no significant difference in CD8 T cells in the percentage of cells positive either for IL-7R (48± 5.09% vs 45.13± 5.18%, Fig 3a) or in the GMFI for MDR septic patients vs CINS patients (33.98± 4.01 vs 38.05±5.47; Fig 3b).

**Fig 4:**
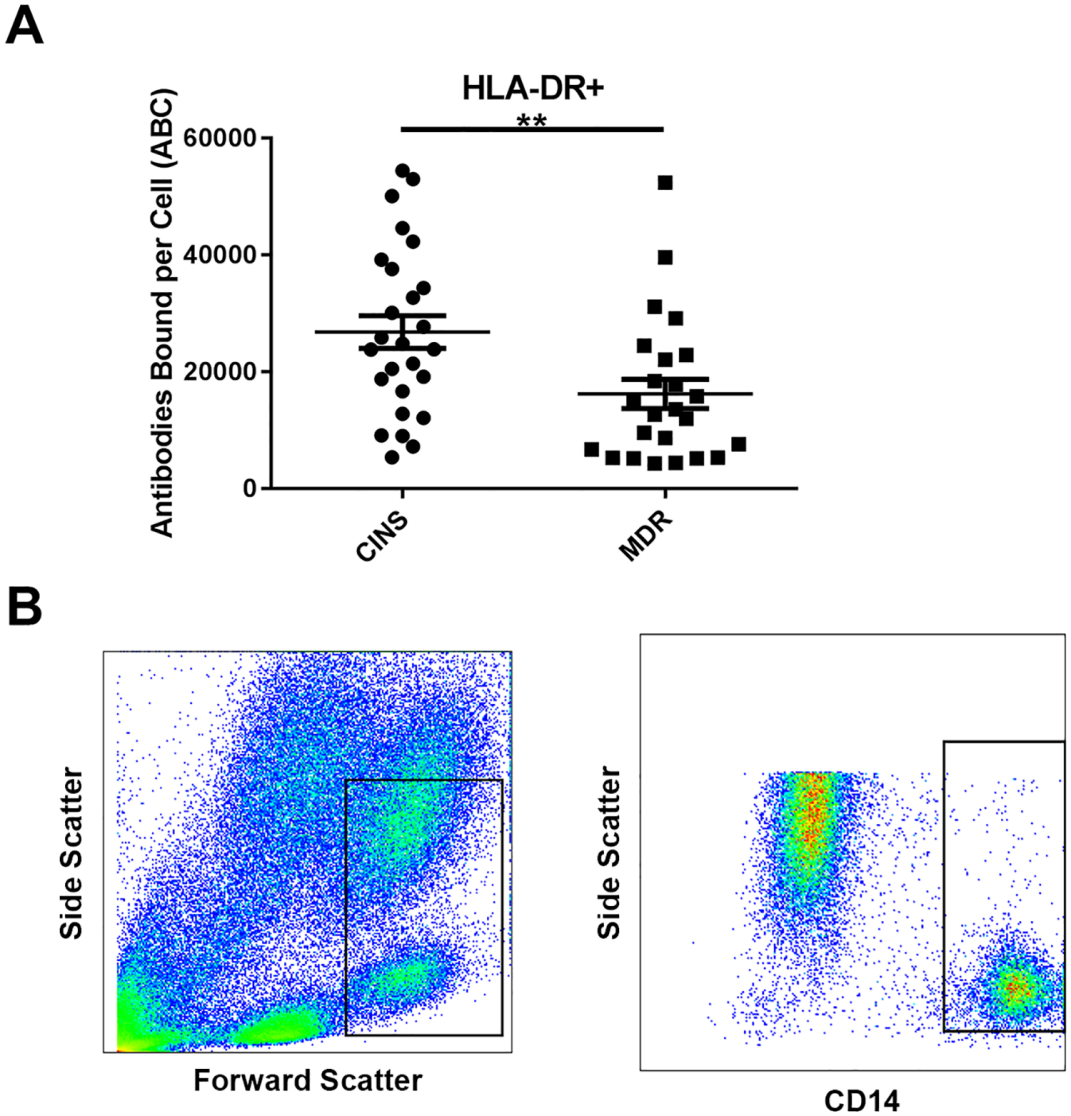
Flow cytometric expression of monocyte HLA-DR. Monocytes in the Multidrug resistant (MDR) Septic patients compared to Critically-Ill Non-Septic (CINS) patients had significantly decreased surface levels of HLA-DR. Gating strategy used for the Quantibrite Anti-HLADR/Anti-Monocyte stain is included (b). ABC = average number of HLA-DR antibodies bound per cell. Bars represent mean +/- S.E.M. **p<0.01.

To evaluate sepsis-induced immunosuppression, we examined monocyte HLA-DR expression. Here, monocyte HLA-DR levels expressed as antibodies bound per cell (ABC) was significantly reduced in patients with sepsis due to MDR pathogens vs CINS patients (16,217.8±2507.4 vs 26,811.4±2809.3, *p* = 0.007; Fig 4A). A representative example of the gating strategy employed in quantitation of monocyte HLA-DR expression is shown in Fig 4B.

**Fig 5:**
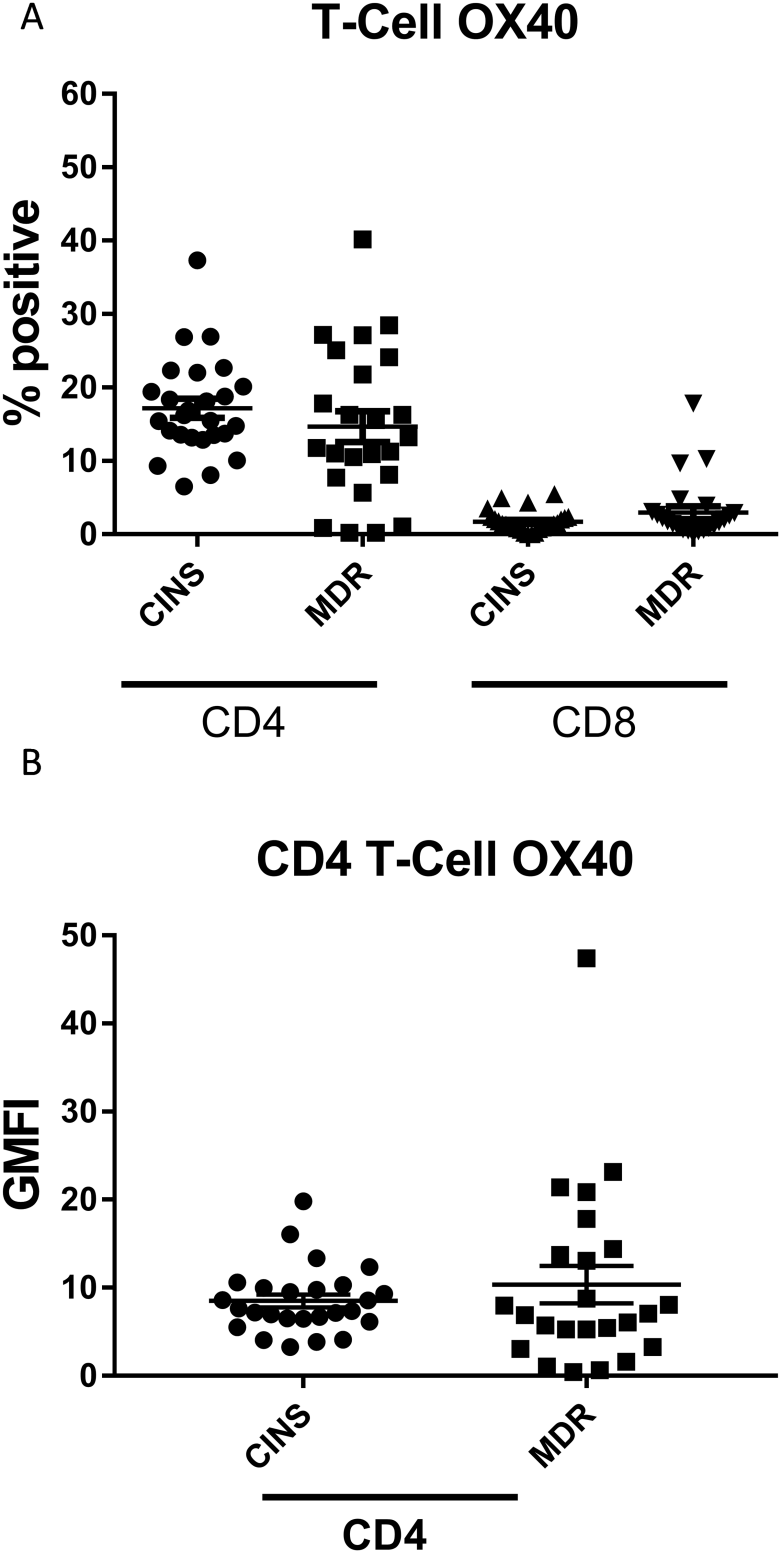
OX-40 surface levels on T cells. There was no difference in OX-40 surface levels in the Multidrug resistant (MDR) Septic patients compared to Critically-Ill Non-Septic (CINS) patients in either the percent of positive cells (a) or receptor density (b). Staining on CD8 T cells was no greater than background.

Finally, to examine markers of activation, we quantified the levels of OX-40 on CD4 and CD8 T cells. The percentage of CD4 T cells expressing OX-40 was not significantly different between MDR septic and CINS patients (14.69± 2.1% and 2.95± 0.85% vs 17.18± 1.3% and 1.72± 0.28%, Fig 5, Table 3). Similarly, there was no difference in the GMFI for OX-40 on CD4 T cells between either cohort (10.35±2.11 vs 8.51±0.74, Fig 5). GMFI for OX-40 on CD8 T cells was not quantitated due to weak staining of the marker on these particular cells.

### ELISpot–IL-7, anti-PD-L1, and OX-40L increase T cell IFN-γ production

**Fig 6:**
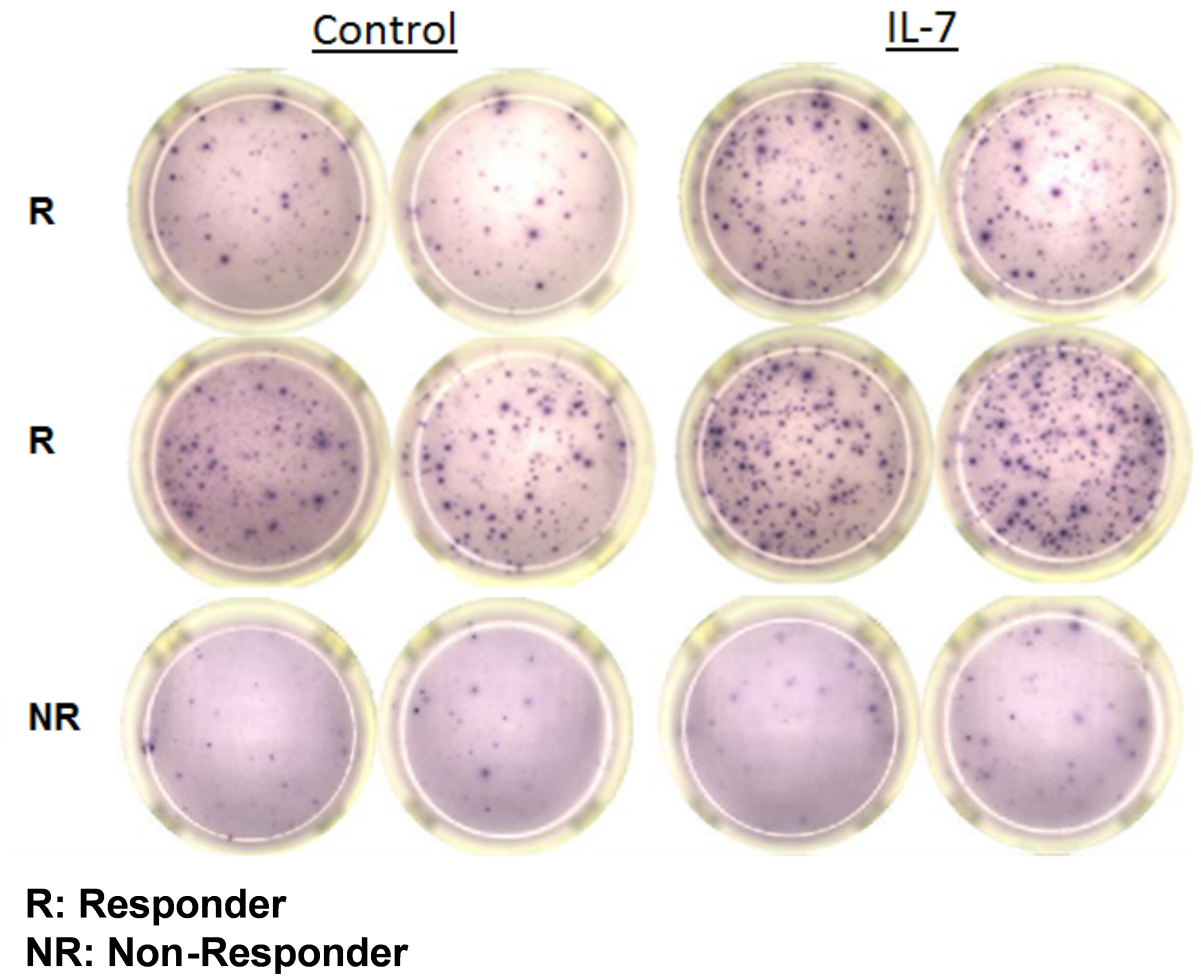
Representative ELISpot wells. Samples displaying a response to treatments showed a greater than 20% increase in the number of IFN-γ producing cells (spots on the membrane). Here, ELISpot images from two representative IL-7 responders (R) are displayed along with a non-responder (NR).

**Fig 7:**
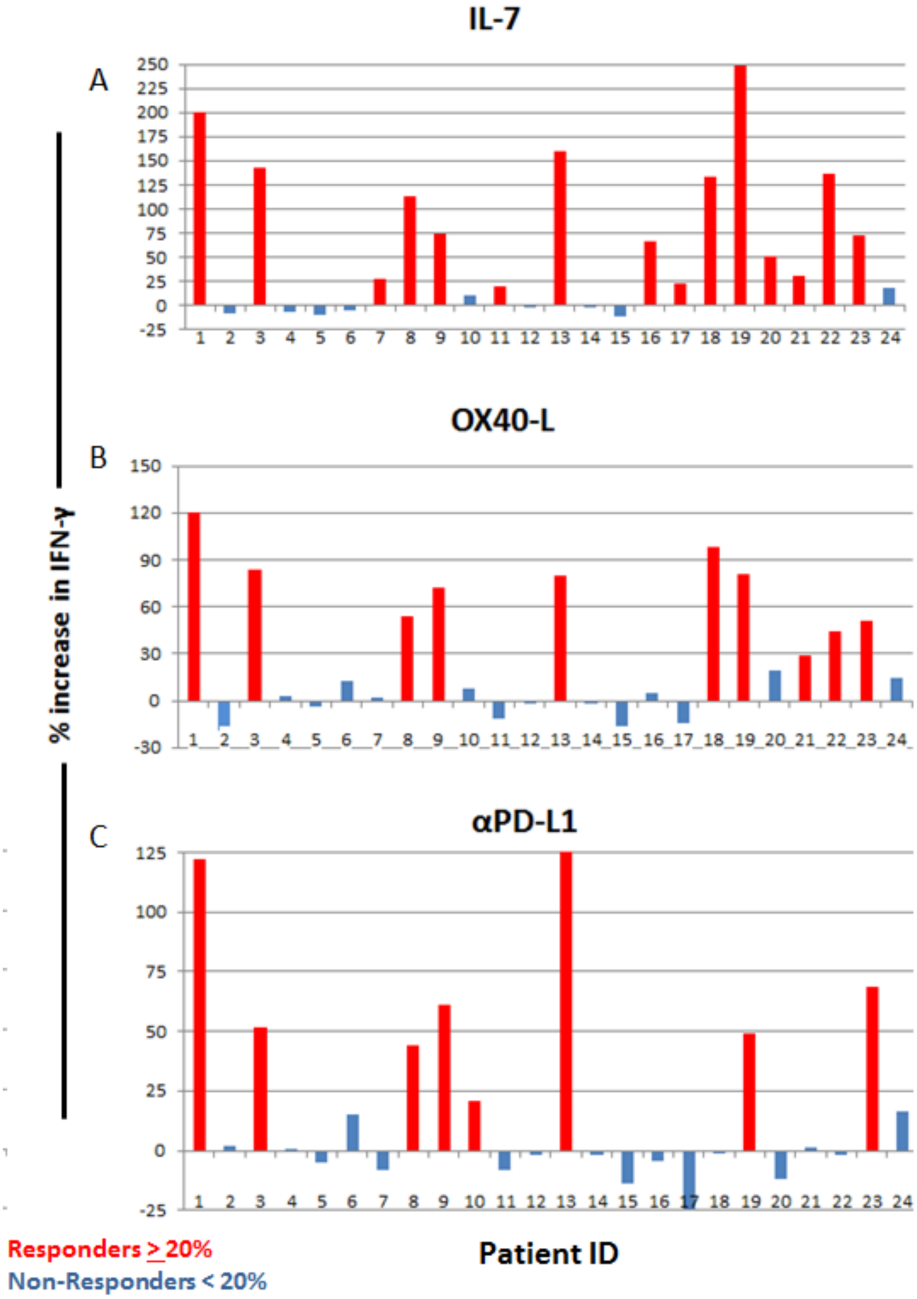
Multidrug resistant (MDR) septic patients’ response to immuno-adjuvant treatments. Patients who displayed an increase in spot numbers of 20% or more in response to treatment were considered responders (Red), while patients who had an increase in spot number of less than 20% were considered non-responders (Blue). 15 of 24 patient samples responded to IL-7 (a), 10 of 24 responded to OX-40L (b), and 8 of 24 responded to PD-L1 antibody (c). Percent increase in IFN-γ was calculated by dividing the number of spots with treatment by the number of spots from sham (Isotype control) treatment.

**Fig 8:**
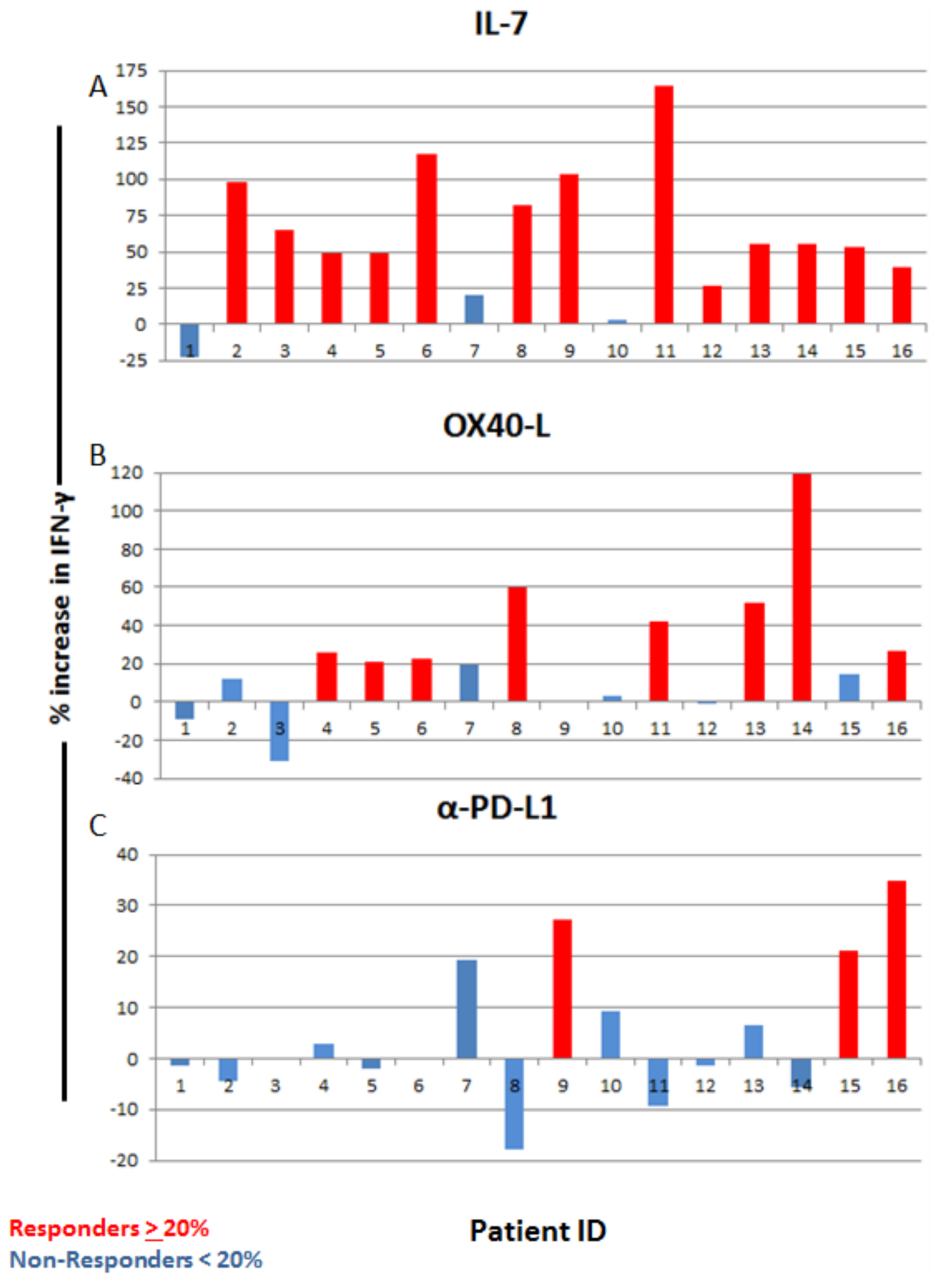
Critically-Ill Non-Septic (CINS) patients’ response to immuno-adjuvant treatments. Patients who displayed an increase in spot numbers of 20% or more in response to treatment were considered responders (Red), while patients who had an increase in spot number of less than 20% were considered non-responders (Blue). 13 of 16 patient samples responded to IL-7, 8 of 16 responded to OX-40L, and 3 of 16 responded to PD-L1 antibody. Percent increase was calculated by dividing the number of spots with treatment by the number of spots from sham (Isotype control) treatment.

ELISpot assays were conducted to detect the number of IFN-γ producing cells. Using this assay, patients were classified as Responders (R) or Non-Responders (NR) based upon an increase in the number of cells positive for IFN-γ immunostaining of at least 20% compared to the control, i.e., isotype control (Figs 6–8). This increase of 20% was selected as the cutoff to define a positive effect of the immuno-adjuvant on T cell IFN-γ production and based upon previous ELISPOT studies from our laboratory showing typical effects of these agents (data not shown).

Differences in the number of septic patients who responded to the three different immuno-adjuvants to increase IFN-γ are shown (Table 4). IL-7 was the most effective in inducing IFN-γ production. In that regard, 15 of 24 septic patients (62.5%) responded to IL-7 while 10 of 24 septic patients (41.6%) responded to OX-40L treatment and 8 of 24 septic patients (33.3%) responded to anti-PD-L1 (Fig 7). These percentages in responders vs non-responders were not statistically different for the three immuno-adjuvants. To better characterize the relative potency of the 3 different immuno-adjuvants, the percent increase in *the number of IFN-γ* positive spots (representing the number of IFN-γ producing T cells) was compared. Of the responding patients, IL-7, OX-40, and anti-PD-L1 caused a 99.9 ± 17.9, 71.4 ± 8.6, and 68.4 ± 13.6 percent increase in the number of IFN-γ producing T cells. These differences were not statistically significant.

**Table 4:** ELISpot % increase in IFN-γ producing cells after treatment.

	Treatment		
	α-PD-L1	IL-7	OX-40L
Responders	68.39(+/-13.56), n = 8	99.88(+/-17.91), n = 15	71.39(+/-8.63), n = 10
Non-Responders	-3.05(+/-2.49), n = 16	-1.84(+/-3.19), n = 9	-1.15(+/-3.57), n = 14

ELISpot analysis for IFN-γ was also performed on 16 CINS patients. Similar to results for the septic patients, IL-7 caused the largest percentage increase in IFN-γ producing cells, i.e., 13 of 16 patients (81.2%) responded (Fig 8). OX-40L caused 8 of 16 (50%) CINS patients to increase IFN-γ while anti-PD-L1 caused 3 of 16 (18.8%) CINS patients responded. Interestingly, the magnitude of the increase in IFN-γ, i.e., the number of IFN-γ producing cells, was less in the CINS compared to the septic patients, (Table 4).

#### Correlation of mortality and the IFN-γ response to immunotherapy in septic patients

The 90-day mortality for the MDR septic cohort was 37.5% while none of the CINS patients died within this timeframe. Intriguingly, 16 patients had an increase in ELISpot T cell IFN-γ production to any of the three immune-adjuvants, i.e., IL-7, OX-40L, or anti-PD-L1, and only 5 of these 16 responders died by day 90, a mortality of 31.25%. Although not statistically significant, patients without a response to IL-7 were more likely to die than those with a response. Conversely, 8 septic patients had no increase in IFN-γ to any of the 3 immuno-adjuvants and 4 of these 8 patients died by day 90, a mortality of 50%.

## Discussion

The purpose of this study was to examine the ability of three unique immuno-adjuvants with differing mechanisms of action, to restore T cell function in patients with sepsis due to MDR bacteria. Our study identified defects in cellular immunity in septic patients with MDR bacteria as evidenced by upregulation of both the percentage and geo mean fluorescence intensity (GMFI) for the inhibitory receptor PD-1 on CD4 and CD8 T cells and decreased monocyte HLA-DR expression (Figs 2 and 4). In addition, there was a non-statistically significant trend toward decreased IL-7 receptor alpha on CD4 T cells from septic patients vs critically-ill non-septic patients (*p* = 0.10) (Fig 3).

Importantly, all three immuno-adjuvants were effective in enhancing T cell IFN-γ production in various subsets of septic patients. IL-7, which directly activates T cells, innate lymphoid cells, and MAIT cells [23], was the most effective in that 62.5% of septic patients responded by increasing the number of IFN-γ producing T cells (Fig 6, Table 4). Potential reasons for the greater efficacy of IL-7 compared to anti-PD-L1 and OX-40L include the fact that IL-7 receptor is constitutively expressed on CD4 and CD8 T cells while PD-1, which inhibits T cell activation by blocking CD28 signaling and is the receptor for PD-L1 [12], and OX-40, which activates T cells through the NF-κB and PI3 Kinase pathways [27], are induced following cell activation [26–28]. Secondly, IL-7 was expressed on a greater percentage of CD4 and CD8 T cells compared to PD-1 and OX-40 (Figs 3 and 5). Because these 3 immuno-adjuvants work by different mechanisms to increase T cell activation (), it may be possible to improve T cell responsiveness in sepsis by combining the different agents. In fact, combination immunotherapy using drugs that are active at different cellular pathways is the current paradigm in oncology trials with over 100 such trials currently underway.

MDR bacteria are strains of bacteria that are resistant to multiple antibiotics that may typically be clinically deployed for the treatment of a particular class of pathogen. The rise in the number of strains of MDR bacteria is one of the most serious problems presently facing the health care community. It is unlikely that development of newer antibiotics alone will be effective in dealing with MDR bacteria because of the rapid development of bacterial resistance to antibiotics. Limiting the inappropriate use of antibiotics by restricting their use and preventing antibiotic prescribing to livestock will help but will likely be insufficient. Many of the individuals that are infected with MDR bacteria are hospitalized patients, patients who are being cared for in long-term health care facilities, oncology patients, or hemodialysis patients; these patients typically have weakened immune systems and are also frequently exposed to these MDR bacteria [4–8]. The fact that most MDR bacteria do not tend to infect patients with normal immune status underscores the central role of immune competence in preventing these types of bacterial infections.

A potential new therapeutic approach to treating patients with sepsis due to MDR bacteria involves enhancing the patient’s intrinsic immunity by using immuno-adjuvants [29, 30]. Apoptotic death and depletion of CD4 and CD8 T cells and T cell exhaustion are hallmarks of patients with sepsis [31–34]. Animal studies of sepsis involving *P*. *aeruginosa* and *S*. *aureus*, two bacteria classified as superbugs, have shown that IL-7, anti-PD-1/L1, and OX-40 ligand restore T cell IFN-γ production, decrease bacterial load, and improve survival [12, 13]. Ex vivo studies of peripheral blood mononuclear cells from patients with sepsis demonstrate that IL-7 and anti-PD-1 and/or anti-PD-L1 decrease apoptotic cell death and enhance T cell IFN-γ production [35, 36]. Both IL-7 and anti-PD-L1 have been used in patients with various infectious disorders. Patients with JC virus-induced progressive multi-focal leukoencephalopathy experienced clearing of viral levels in the cerebrospinal fluid, improved brain imaging, and resolution of clinical symptoms after treatment with IL-7 [37]. Anti-PD-1 and anti-PD-L1 have been used to treat patients with hepatitis C and HIV, respectively. Both anti-PD-1 and anti-PD-L1 were well-tolerated and showed efficacy by significantly decreasing viral load in a subset of patients. Recently, anti-PD-1 was lifesaving when used in combination with IFN-γ on a compassionate basis in a patient who was dying of disseminated mucormycosis [38]. These benefits of IL-7, anti-PD-1, and anti-PD-L1 immuno-adjuvant therapy in patients with viral and fungal infections support their potential efficacy in patients with MDR bacteria.

An intriguing finding in the present study was the difference in mortality in patients whose peripheral blood mononuclear cells (PBMCs) responded to immuno-adjuvants by increasing IFN-γ production vs patients whose PBMCs were non-responsive to immuno-adjuvants (Table 3). The results of the ELISpot analysis may identify patients whose T cells are irreversibly impaired, and who would likely stand to benefit the most from these therapies. These types of functional studies examining T cell cytokine production could be combined with other data including absolute lymphocyte counts and gene expression data to identify patients with sepsis who are good candidates for immunotherapy. It is also important to note that the failure of the patients’ PBMCs to respond to immuno-adjuvants *ex vivo*, i.e., in the incubation chamber, does not necessarily indicate that these patients will not respond to combination immunotherapy that targets multiple independent immune defects occurring during sepsis.

The present results showing a potent effect of IL-7, OX-40L, and anti-PD-L1 to improve T cell IFN-γ production in patients with MDR bacteria provides additional support for clinical trials of these agents in life-threatening sepsis due to MDR bacteria. A small phase II clinical trial of IL-7 was recently conducted in patients with septic shock, some of whom had MDR bacterial infection in the context of sepsis. IL-7 was safe, induced T cell activation, and increased circulating CD4 and CD8 T cells 3–4 fold [23]. Similarly, an ascending dose phase I trial of anti-PD-L1 in patients with septic shock was recently conducted (NCT02739373). Anti-PD-L1 was well tolerated and there was a trend toward increased monocyte HLA-DR expression with ascending dose of drug (Abstract presented at 2018 Society of Critical Care Medicine). Although OX-40 ligand has not yet been tested clinically in sepsis, there are currently over 10 clinical trials of OX-40 ligand in oncology. OX-40 ligand has been well-tolerated and shown clinical efficacy in a subset of cancer patients [39]. Given the remarkable similarities in the immunosuppressive mechanisms active in both disorders [15] and the excellent safety profile of OX-40 ligand, we believe that OX-40 ligand should also be investigated as a potential immuno-adjuvant in patients with sepsis due to MDR bacteria.

There are several limitations to this study. First, our study design allows for only a single time point measurement of immune function and does not take into account either the preceding or subsequent disease course. Although this snapshot in time is useful in understanding response to the three immuno-adjuvants, it may not necessarily represent dynamic response in disease course, nor potential septic patient immune phenotypes prior to presentation to the ICU. Secondly, this study only includes a control of critically-ill, non-septic surgical patients. The addition of critically-ill, non-multidrug resistant bacterial sepsis controls or healthy controls would improve value in understanding specific immune response to each of the immuno-adjuvants. Finally, there were significant clinical differences in the septic versus critically ill, non-septic patients. The severity of illness in the septic patients was greater than that for the critically ill, non-septic patients. Approximately 30% of septic patients required inotropic agents, i.e. norepinephrine and/or epinephrine for treatment of shock while only one critically ill, non-septic patients required such therapy. Septic patients also had more organ dysfunction than the critically ill, non-septic patients. Therefore, direct comparison between the T-cell response of the septic and critically ill, non-septic patients to the 3 immune-adjuvant therapies has significant limitations.

In conclusion, patients with sepsis due to MDR bacteria display an immunophenotype consistent with T cell exhaustion and monocyte suppression. Immuno-adjuvant therapy with three mechanistically distinct agents increased T cell production of IFN-γ, a cytokine critical for host antimicrobial defense. Given the remarkable success of immunotherapy in oncology, the similarities in the immune defects in sepsis and cancer, and the high mortality in patients with sepsis due to MDR bacteria, therapeutic trials to enhance host immunity should be a top priority. Immunotherapy offers a potential way forward that meets public health demands of newer, more innovative therapies that target the underlying cause of MDR infections: impaired immunity.

## Supporting information

S1 Table Antibodies. (DOCX)
