# Supplementary material for: Restoration of T Cell function in multi-drug resistant bacterial sepsis after interleukin-7, anti-PD-L1, and OX-40 administration
Source: PLoS One. 2018 Jun 26;13(6):e0199497. doi: 10.1371/journal.pone.0199497 (PMC6019671; doi:10.1371/journal.pone.0199497)
Supplement: S1 Table — (DOCX) [file pone.0199497.s001.docx]

**S1 Table**

|  | **Antibodies** |  |
| --- | --- | --- |
| Antibody | Vendor | Catalog number |
| CD8 | BioLegend | 344714 |
| CD4 | BioLegend | 300530 |
| CD3 | BioLegend | 300440 |
| OX-40 | BioLegend | 350008 |
| CD279(PD-1) | BioLegend | 329906 |
| CD127 | BioLegend | 351316 |
| CD14 | BioLegend | 301824 |
| CD15 | BioLegend | 323004 |
| CD274(PD-L1) | BioLegend | 329706 |
| Quantibrite Anti-HLA-DR/Anti-Monocyte | BD | 340827 |
